# Liposuction of Breast Cancer-Related Arm Lymphedema Reduces Fat and Muscle Hypertrophy

**DOI:** 10.1089/lrb.2020.0120

**Published:** 2022-02-28

**Authors:** Tobias Karlsson, Magnus Karlsson, Karin Ohlin, Gaby Olsson, Håkan Brorson

**Affiliations:** ^1^ Department of Clinical Sciences, Lund University, Malmö, Sweden.; ^2^ Clinical and Molecular Osteoporosis Research Unit, Department of Orthopedics, Skåne University Hospital, Malmö, Sweden.; ^3^ Department of Plastic and Reconstructive Surgery, Skåne University Hospital, Malmö, Sweden.

**Keywords:** breast cancer, lymphedema, liposuction, muscle, fat, DXA

## Abstract

***Background:*** Adipose tissue deposition is a known consequence of lymphedema. A previous study showed that the affected arm in patients with nonpitting breast cancer-related lymphedema (BCRL) had a mean excess volume of 73% fat and 47% muscle. This condition impairs combined physiotherapy as well as more advanced microsurgical methods. Liposuction is, therefore, a way of improving the effects of treatment. This study aims to evaluate the tissue changes in lymphedematous arms after liposuction and controlled compression therapy (CCT) in patients with nonpitting BCRL.

***Methods and Results:*** Eighteen women with an age of 61 years and a duration of arm lymphedema (BCRL) of 9 years were treated with liposuction and CCT. Tissue composition of fat, lean (muscle), and bone mineral was analyzed through dual energy X-ray absorptiometry (DXA) before, and at 3 and 12 months after surgery. Excess volumes were also measured with plethysmography. The median DXA preoperative excess volume was 1425 mL (704 mL fat volume, 651 mL lean volume). The DXA excess volume at 3 months after surgery was 193 mL (−196 mL fat volume, 362 mL lean volume). At 12 months after surgery, the median excess DXA volume was 2 mL (−269 mL fat volume, 338 mL lean volume). From before surgery to 3 months after surgery, the median DXA excess volume reduced by 85% (*p* < 0.001) (fat volume reduction 128% (*p* < 0.001), lean volume reduction 37% (*p* = 0.016)). From before surgery to 12 months after surgery, it reduced by 100% (*p* < 0.001) (fat volume reduction 139% [*p* < 0.001], lean volume reduction 54% [*p* = 0.0013]).

***Conclusions:*** Liposuction and CCT effectively remove the excess fat in patients with nonpitting BCRL, and a total reduction of excess arm volume is achievable. A postoperative decrease in excess muscle volume is also seen, probably due to the reduced weight of the arm postoperatively.

## Introduction

Surgical treatment of breast cancer is a known cause of arm lymphedema.^1^ The incidence of breast cancer-related lymphedema (BCRL) ranges from 4% to 65% in different studies, depending on whether axillary clearance and radiotherapy were performed.^2–4^ High body mass index (BMI), mastectomy, chemotherapy, and lack of physical activity are other factors associated with this condition.^1,5^ The mechanism behind BCRL involves trauma to the lymphatic vessels when the lymph nodes are removed, potentially accentuated by scarring after radiotherapy. This leads to lymph stasis with interstitial edema in the subcutaneous tissue. The accumulation of fluid leads to chronic inflammation, resulting in fat deposition.^6–11^ The mechanism behind the change in tissue composition in lymphedema with more adipose tissue is not clear. One theory is that the accumulated lymph attracts inflammatory cells, including macrophages, and these, in turn, release different mediators that stimulate adipogenesis.^7,8^ The result is a lymphedema consisting mostly of adipose tissue, and lymph that shows pitting when pressure is applied by pressing the thumb on the edematous tissue for 1 minute (pitting test).^12,13^

Methods for measuring soft-tissue composition include dual energy X-ray absorptiometry (DXA),^14–18^ bioimpedance analysis,^17–19^ computed tomography^11^ and magnetic resonance imaging (MRI).^10^ Of these, DXA is one of the clinically most usable methods due to its wide availability, low radiation dose, and short time requirement. DXA has also been proved to measure soft-tissue composition accurately and with great repeatability.^20^ A previous study conducted at the Department of Plastic and Reconstructive Surgery at Skåne University Hospital, where preoperative DXA scans were made of the arms of 18 women with BCRL, showed a 73% increase in excess volumes of fat, 47% in muscle, and 7% in bone mineral.^9^

The first-line treatment for lymphedema is conservative treatment known as complex (or complete) decongestive therapy (CDT). CDT includes bandaging, skin care, compression garments, physical activity, and manual lymphatic drainage, with the aim of removing the fluid part of the excess volume.^21,22^ Compression garments and skin care are involved in the treatment known as controlled compression therapy (CCT). CCT is usually practiced in combination with liposuction to treat late-stage nonpitting lymphedemas.^23,24^

In untreated lymphedemas, CCT alone is able to reduce the excess volume to 47%,^23^ but this therapy cannot remove the adipose tissue. The same applies to methods of microsurgery such as lymph node transfers,^25–27^ lymphovenous shunts,^28,29^ and lymph vessel transplantation.^30,31^ All these surgical methods aim to increase lymph drainage but cannot remove adipose tissue. Liposuction, in contrast, is able to remove excess adipose tissue, leading to complete reduction of the excess volume in both upper limb lymphedema^24,32,33^ and lower limb lymphedema.^34,35^

The excess muscle mass and bone mineral mass in the lymphedematous arm might be a result of the increased weight on the arm caused by the lymphedema, promoting an enlargement of muscle cells,^36^ osteoblast activity, and bone mineral deposition.^37^ Long-term follow-up of patients treated with liposuction in combination with CCT for BCRL shows a complete reduction of the excess volume of the edematous arm after 5 years.^24^ Before a steady state is reached, regular volumetric measurements show a continuous decrease in excess volume in the limb. This takes place naturally as the fat is removed, and hypothetically through a continuous reduction of muscle, and to a minor extent bone mineral mass, brought about by the reduced weight load.

To our knowledge, no prospective study has assessed changes in soft-tissue composition after liposuction for arm lymphedema using DXA; however, this has been made using MRI. One study included five patients with leg lymphedema and reported 4% median decrease of muscle volume after liposuction with follow-up ranging from 5 to 12 months.^38^ Two other studies have reported on the same 13 patients with lymphedema (seven arms and six legs) and found a significant decrease in subfascial muscle/water volume 1 year after liposuction, but no absolute numbers were presented.^6,39^

This study aims to follow soft-tissue composition 1 year after the operation in the 18 patients with BCRL for whom preoperative data were previously reported.^9^ The follow-up will focus on changes in fat and muscle volume, but also in bone mineral volume. Furthermore, the total limb volume obtained with DXA will be analyzed for correlation with plethysmography (PG), a volume-measurement method also used in this study, which is currently viewed as the gold standard in volume assessments.

## Materials and Methods

### Patients

Eighteen consecutive patients with BCRL and a median age of 61 years (interquartile range [IQR] 54–72) underwent liposuction. They had previously been studied through DXA measurements.^9^ Inclusion criteria involved a lymphedema with >10% increase of volume compared with the nonlymphedematous arm (excess volume), patients who were willing to undertake DXA measurements, minimal or nonpitting edema of the affected arm (<5–6 mm), no surgical implants or prostheses that could interfere with DXA measurements, and acceptance of continued CCT. An excess volume of >10% in normal arms is considered to be a severe increase in total volume, even in dominant compared with nondominant arms.^40^

Exclusion criteria involved lymphedema in both arms, active cancer, and ongoing erysipelas. Of 18 patients, 16 had undergone modified radical mastectomy and 2 had undergone partial mastectomy with axillary lymph node dissection. In addition, 15 patients were subsequently treated with radiotherapy and four patients with chemotherapy. All lymphedemas were treated conservatively before liposuction until no further reduction in volume could be seen and pitting was, therefore, minimal.

### Measurements

#### Arm volumes

PG, a water-displacement method widely used for measuring lymphedematous limbs, is a reliable technique and the gold standard for measuring volume.^41–43^ The arm is lowered into a container of water, and the overflow of water is collected and weighed to the nearest 5 g, corresponding to 5 mL of volume. The areas of measurement first include the hand and a 40 cm section of the arm measured from the ulnar styloid process (for comparison with DXA), and second, the whole arm up to the axilla (for comparison with aspirated volumes). Volumes were measured preoperatively, and after 3 and 12 months. Measurements were taken by the same physiotherapist (G.O.) and occupational therapist (K.O.).

#### Dual energy X-ray absorptiometry

Body composition was measured with DXA total body scan (DPX-L version 3.2; Lunar, Madison, WI) preoperatively, and after 3 and 12 months. Scans were performed with the patient in the supine position with the arm slightly abducted, and with foam tucked in between the arm and torso to avoid any contact with the body to minimize calculation errors. Both arms were analyzed at each instance. Regions of interest (ROI) were analyzed distally to include the hand, and proximally to a point 40 cm from the ulnar styloid process, a ROI equivalent to the volume measured by PG. Standard Lunar whole-body software was used to analyze fat mass, lean body mass (LBM), and bone mineral content (BMC). LBM consists predominantly of muscle but includes all tissue elements except fat and bone.

An estimation was made that the excess LBM in the lymphedematous arm compared with the nonaffected arm predominantly consisted of hypertrophied muscle. Thus, excess lean mass is referred to as excess muscle mass. The following ratios were used to convert the measured weight into tissue volume: fat 0.92 g/mL,^44^ muscle 1.06 g/mL,^45–47^ and bone 3.15 g/mL.^48^ One technician carried out all DXA scans and one researcher (H.B.) conducted all the software analyses for the DXA measurements. The DXA machine was calibrated daily during the study period.

#### Liposuction and CCT

The liposuction technique has been described in detail in previous studies.^24^ Two weeks before surgery, measurements were taken for custom-made garments, using the normal arm as a template. Three garments were ordered (Elvarex^®^, compression class 2; Essity, Sweden) and one was sterilized to be placed on the arm during surgery. The sterilized garment was used for only 2 days, since sterilization slightly decreases compression. In short, power-assisted liposuction (Lipomatic, Nutational Infrasonic Liposculpture^®^; Euromi, Andrimont, Belgium) was performed with a bloodless field, using a tourniquet placed proximally on the upper arm. Around 10 incisions of 4–5 mm were made along the arm, and liposuction was performed using 15 cm long cannulas with a diameter of 3 and 4 mm.

A sterile tape measure was used to achieve the same circumferences as in the contralateral arm. A sterile compression sleeve was then applied on the arm up to the tourniquet, which was subsequently removed. Liposuction of the remaining proximal part of the arm and shoulder region was then performed using the tumescence method, where 1000 mL of saline solution mixed with 1 mL adrenaline and 20 mL lidocaine 2% (Xylocaine^®^; AstraZeneca PLC, London, United Kingdom) was injected subcutaneously. The aspirate was collected in canisters of 2000 mL, where portions of fat and fluid were measured as a matter of routine after 24 hours sedimentation at room temperature.

Antibiotics were administered during and after surgery with isoxazolylpenicillin intravenously during the first 24 hours and thereafter with tablets during the following 10 days. Clindamycin was used if the patient was allergic to penicillin. Garments were changed at 2 and 4 days after surgery, in conjunction with showering and lubrication of the skin with lotion. The patient was discharged after the second change of garments on the 4th postoperative day. The first week after discharge, garments were changed every second day. Thereafter, garments were changed every day. Garments were washed after each change to be used the next day. Excess volume was measured, and new sets of garments were ordered as required, at 1, 3, 6, 9, and 12 months. Garments were worn continuously. Liposuction was performed by the same surgeon (H.B.) in all patients.

### Statistics

The analysis was conducted using IBM SPSS Statistics for MacOS 10.14, Version 25.0. Armonk, NY: IBM Corp, released 2017. The normal distribution of the data was tested using the Shapiro–Wilk test, but a significant difference was found in the spread of the values in some of the variables compared with a normal distribution. Thus, values are presented as median and IQR unless otherwise stated. Associations between pre- and postoperative volumes were tested with the Wilcoxon matched-pairs signed-rank test. A *p*-value of <0.05 was considered statistically significant. Correlations between the two methods of measurement (DXA and PG) were analyzed in a Bland–Altman^49^ scatter plot. This was possible since the differences between within-subject measurements for the lymphedematous arm followed the normal distribution.

The significance observed between the two methods of measurement was assessed by calculating 95% confidence intervals (95% CI) for the mean differences. All statistical analyses were performed by the same researcher (T.K.).

### Ethics

Approval for this study was obtained from the Ethics of Human Investigation Committee of Lund University (697 98), as well as the Radiation Protection Committee of Skåne University Hospital in Malmö (981212). Written acceptance was received from all participants in the study, and it was planned and executed in agreement with the concepts contained in the Declaration of Helsinki of 1964, and its 2008 revision.

## Results

Patient characteristics are presented in Table 1. In general, the cohort was composed of women with a median age of 61 (IQR: 54–72) at liposuction. The median time from cancer surgery until debut of lymphedema was 1 year (IQR: 0.3–2.8), and the median duration of lymphedema was 9 years (IQR: 5–17) until liposuction was performed. All patients exhibited late-stage II-III lymphedemas according to the International Society of Lymphology classification scale.^50^ The initial median BMI was 28.1 kg/m^2^ (IQR: 26.6–30.3), at 3 months BMI was 27.8 kg/m^2^ (IQR: 26.6–30.1), and after 1 year BMI was 27.7 kg/m^2^ (IQR: 25.9–29.0), and do not represent a significant change during follow-up (*p* = 0.22 and *p* = 0.13, respectively) (Table 1).

**Table 1.** Demographics of the 18 Patients

Demographics		Percent
BMI, median (IQR)		
Preoperatively	28.1 (26.6–30.3)	
3 months	27.8 (26.6–30.1)	
1 year	27.7 (25.9–29.0)	
Age at cancer surgery, median (IQR)	49 (41–55)	
Interval between cancer surgery to debut of lymphedema (years), median (IQR)	1 (0.3–2.8)	
Duration of lymphedema (years), median (IQR)	9 (5–17)	
Age at investigation/liposuction (years), median (IQR)	61 (54–72)	
Interval between cancer surgery and investigation (years), median (IQR)	13 (6–19)	
Surgery, *n*		
Radical mastectomy	16	89
Partial mastectomy	2	11
Chemotherapy, *n*		
Yes	4	22
No	14	78
Radiotherapy, *n*		
Yes	15	83
No	3	17
CDT, *n*		
Yes	16	89
No	2	11
Compression pumping, *n*		
Yes	13	72
No	5	28
Preoperative use of compression garments, *n*		
Yes	18	100
No	0	0

Values are presented as number and percentage unless otherwise stated.

BMI, body mass index; CDT, complex (or complete) decongestive therapy; IQR, interquartile range.

### Liposuction

**FIG. 1.**
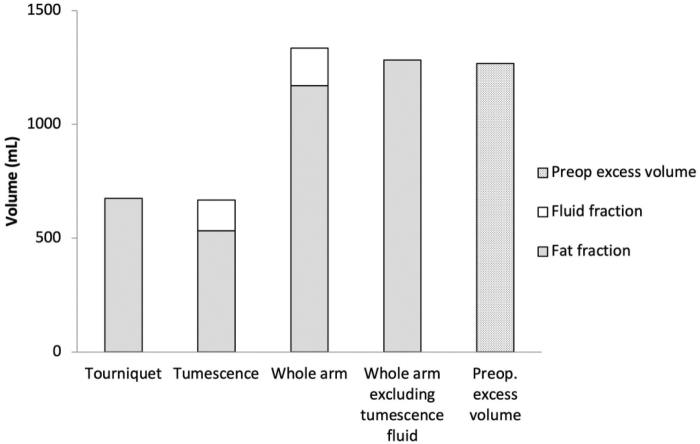
Aspirate analysis in 18 patients (median, IQR) (fat and fluid fractions) and excess volumes. The excess volume was 1268 mL (IQR 1090–1748). Tourniquet: Fractions removed while using the tourniquet. Tumescence: Fractions removed from the area where the tourniquet was applied. Whole arm: Fractions of the total aspirate. Whole arm excluding tumescence fluid: The volume of all fat + fluid fractions when using tourniquet. Preoperative excess volume: Volume of lymphedematous arm minus volume of normal arm, both arms including also the part of the arm above 40 cm, from the wrist, up to the axilla. The volume of aspirate removed when a tourniquet was applied was 728 mL (IQR: 553–905) and contained 100% (IQR: 92–100) fat. The volume of aspirate removed with tumescence was 683 mL (IQR: 543–828), and the proportion of fat was 76% (IQR: 60–91). The high proportion of fluid in the tumescence fraction was due to the aspirated tumescent fluid. Thus, excluding the fluid in the tumescence fraction gives an aspirate fat content of 1283 mL (IQR: 1021–1658), and a 100% proportion of fat (IQR: 94–100). IQR, interquartile range.

Liposuction was performed with a median total aspirate volume (tissue+fluid) of 1323 mL (IQR: 1230–1828). The median volume of aspirate removed when a tourniquet was applied was 728 mL (IQR: 553–905), and consisted of median 100% (IQR: 92–100) fat. When the tumescence portion of the aspirate was included, with a median volume of 683 mL (IQR: 543–828), but the fluid in the tumescence fraction (median 135 mL [IQR: 63–330]) was excluded, the median total aspirate fat content was 100% (IQR: 94–100), as illustrated in Figure 1. The median total amount of fat aspirated was 1170 mL (IQR: 1021–1624). The percentage of fat when the tumescence fluid fraction was included was 85% (IQR: 80–94).

### Tissue reduction over 1 year after liposuction

The preoperative excess volume, measured with DXA and PG, was 1425 mL (IQR: 1049–1538) and 1213 mL (IQR: 1014–1676), respectively. This represents an excess volume of 60% (IQR: 49–65) with DXA measurements and 46% (IQR: 36–58) with PG measurements.

A substantial reduction could be seen 3 months after liposuction, where the excess volume measured with DXA and with PG was 193 mL (IQR: 81–295) and 165 mL (IQR: 70–233), respectively. In the 3 months after surgery, this represents a median decrease in total excess volume of 1207 mL (IQR: 986–1395) (*p* < 0.001) or 85% (IQR: 80–94) measured with DXA, and 1168 mL (IQR: 790–1475) (*p* < 0.001) or 88% (IQR: 77–92) measured with PG.

**FIG. 2.**
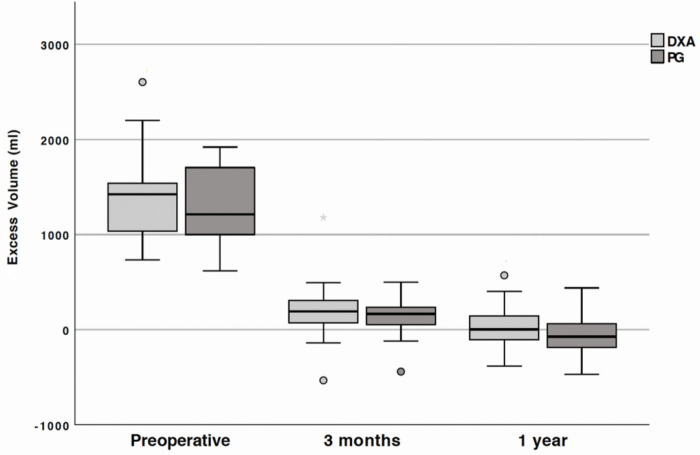
Preoperative excess total volume (mL) before and after surgery, measured with DXA and PG. The *line within the box* represents median excess volumes, *box length* represents the IQR, and *whiskers* represent the lowest and highest values except outliers (*circles*) and extreme outliers (*stars*). Reductions measured with both DXA and PG were statistically significant at 3 months (*p* < 0.001 and *p* < 0.001, respectively) and 1 year (*p* < 0.001 and *p* < 0.001, respectively). DXA, dual energy X-ray absorptiometry; PG, plethysmography.

In the follow-up after 1 year, the excess median volume was 2 mL (IQR: −90 to 139) estimated with DXA, and a complete reduction of −73 mL (IQR: −180 to 59) estimated with PG. This represents a median decrease of 1460 mL in total excess volume (IQR: 1093–1675) (*p* < 0.001) or 100% (IQR: 87–106) measured with DXA, and 1380 mL (IQR: 1144–1636) (*p* < 0.001) or 106% (IQR: 97–115) measured with PG (Fig. 2).

**FIG. 3.**
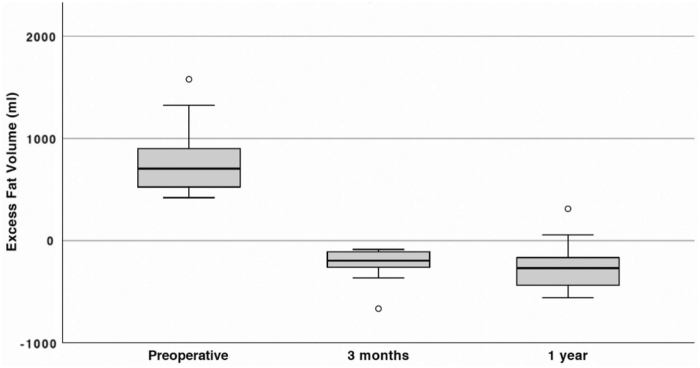
Excess fat tissue volume (mL) before and after surgery. The *line within the box* represents median excess volumes, *box length* represents the IQR, and *whiskers* represent the lowest and highest values except outliers (*circles*). Values obtained with DXA, *n* = 18. The median volume reductions from preoperative to 3 months, as well as preoperative to 1 year, show statistical significance (*p* < 0.001 and *p* < 0.001, respectively).

**FIG. 4.**
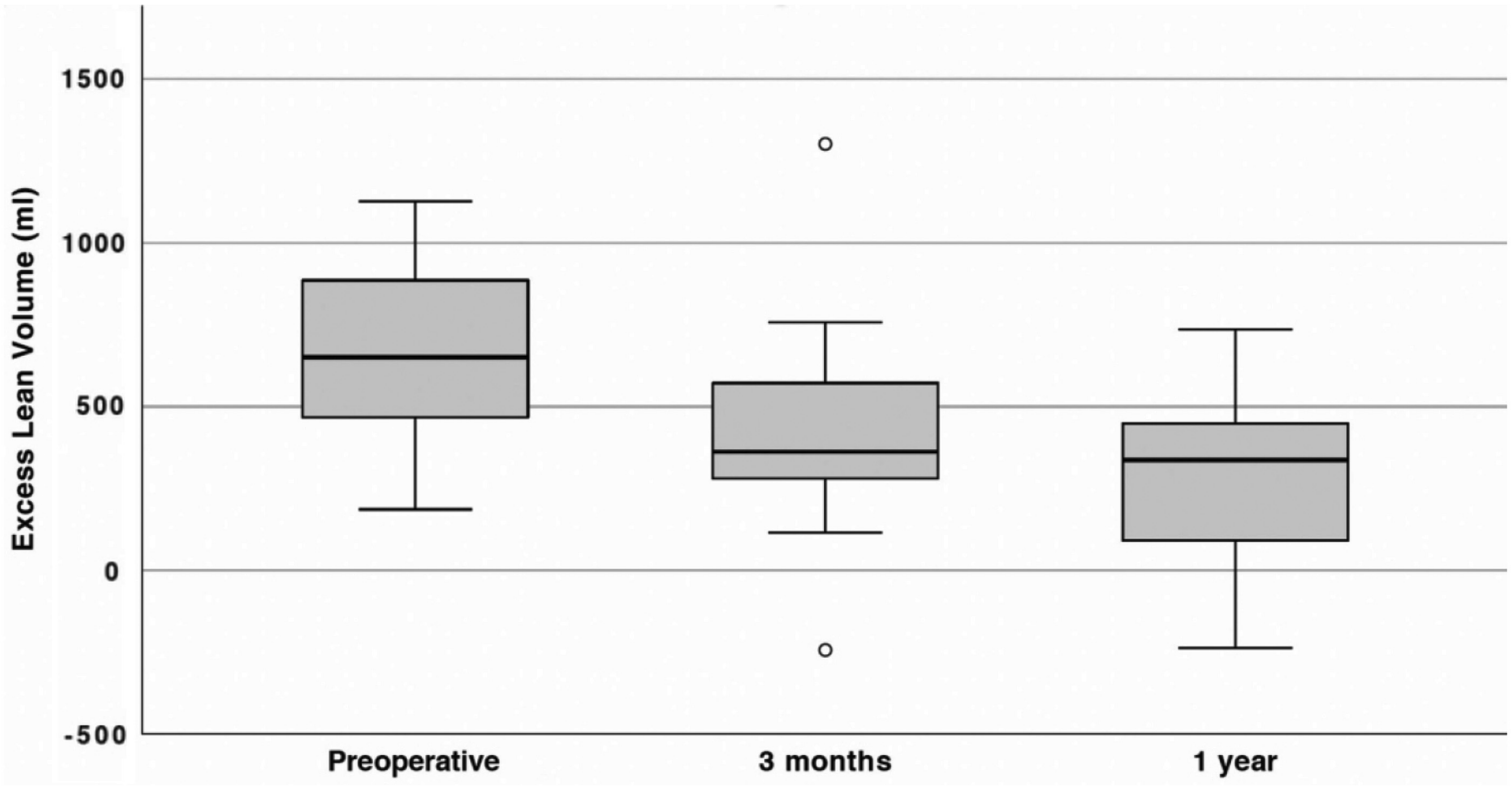
Excess lean tissue volume (mL) before and after surgery. The *line within the box* represents median excess volumes, *box length* represents the IQR, and *whiskers* represent the lowest and highest values except outliers (*circles*). Values obtained with DXA, *n* = 18. The median volume reductions from preoperative to 3 months, as well as preoperative to 1 year, show statistical significance (*p* = 0.016 and *p* = 0.0013, respectively).

**FIG. 5.**
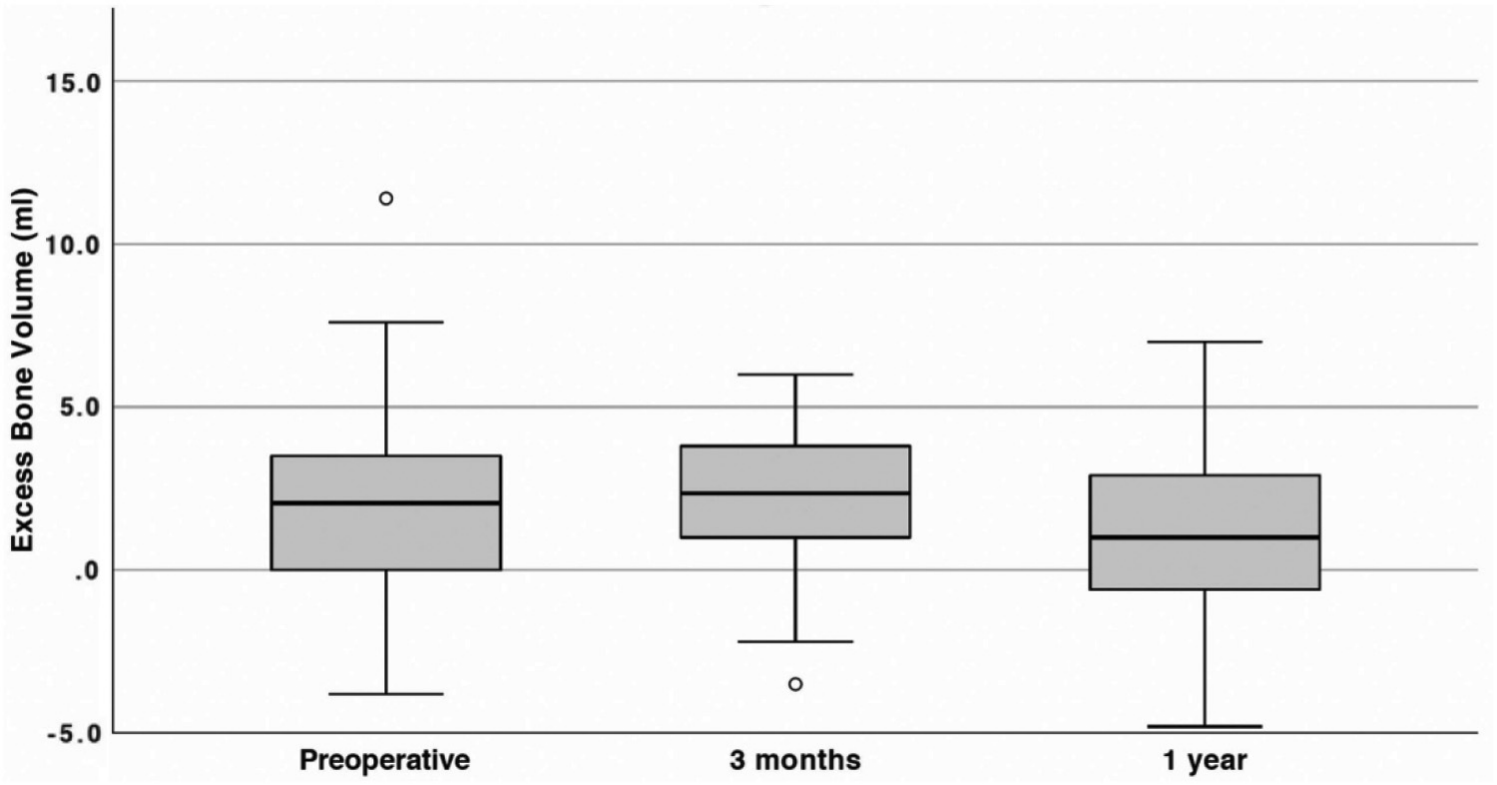
Excess bone mineral volume (mL) before and after surgery. The *line within the box* represents median excess volumes, *box length* represents the IQR, and *whiskers* represent the lowest and highest values except outliers (*circles*). Values obtained with DXA, *n* = 18. The change in median volume from preoperative to 3 months did not show statistical significance (*p* = 0.98), but the reduction from preoperative to 1 year was statistically significant (*p* = 0.045).

Figures 3–5 illustrate DXA measurements of fat volume, lean volume (muscle), and BMC preoperatively, after 3 months and after 1 year.

### Changes in fat volume

A total reduction in excess fat mass was seen as early as 3 months postoperatively, with a reduction in the median excess fat volume from 704 mL (IQR: 545–885) preoperatively to −196 mL (IQR: −258 to −112) after 3 months and −269 mL (IQR: −420 to −166) after 1 year.

This represents a significant median reduction in excess fat volume of 910 mL (IQR: 800–1050) (*p* < 0.001) or 128% (IQR: 116–134) from before surgery to 3 months after surgery. The reduction from before surgery to 1 year after surgery was 929 mL (IQR: 724–1336) (*p* < 0.001), corresponding to a 139% decrease in fat volume (IQR: 125–151) (Fig. 3). The reason the total volume of adipose tissue aspirated at liposuction (median 1170 mL [IQR: 1021–1624]) was higher than preoperative excess adipose tissue volume measured with DXA (704 mL [IQR: 545–885]) is that the shoulder part of the arm was also treated, whereas DXA measures the hand and the arm from 40 cm proximal to the ulnar styloid process.

### Changes in muscle volume

There was a significant decrease in muscle volume postoperatively. Figure 4 shows excess lean volume (predominantly muscle) compared with the healthy arm. A preoperative median lean volume excess of 651 mL (IQR: 475–880) decreased to 362 mL (IQR: 288–549) after 3 months and to 338 mL (IQR: 104–446) after 1 year. This corresponds to a median decrease of 205 mL (IQR: 11–378) (*p* = 0.016) or 37% (IQR: 2–57) from before surgery to 3 months after surgery, and 352 mL (IQR: 160–608) (*p* = 0.0013) from before surgery to 1 year after surgery, which represents a decrease of 54% (IQR: 24–83) in muscle volume.

### Changes in bone mineral volume

A decrease in BMC was also seen postoperatively at the 1-year follow-up, but not at the 3-month visit. The preoperative excess BMC of 2.1 mL (IQR: 0.4–3.4) increased to 2.4 mL (IQR: 1.1–3.7) after 3 months, but decreased to 1.0 mL (IQR: −0.5 to 2.7) after 1 year, representing a statistically significant decrease of 1.1 mL (IQR: 0.3–1.9) or 36% (IQR: −26 to 81) after 1 year (*p* = 0.045), but there was no statistically significant difference after 3 months (*p* = 0.98) (Fig. 5).

### PG versus DXA

**FIG. 6.**
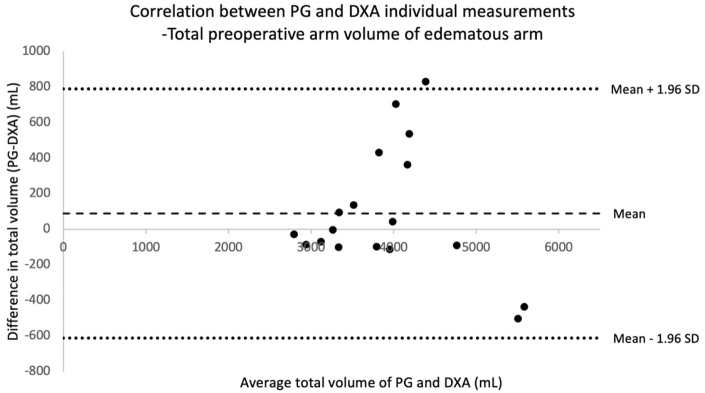
Bland–Altman correlation table showing correlation between total arm volumes in the lymphedematous arm preoperatively before liposuction, measured with DXA and PG. Each point in the diagram represents one patient and two volume measurements, one with DXA and the other with PG. On the x-axis is the mean volume of the two measurements, and the y-axis represents the difference between the two measurements. The area between the two *dotted lines* represents the mean difference within 1.96 SD above and below the mean difference (*dashed line*). All measurements are in milliliters (mL). (mean = 89 mL [95% CI: *−*89 to 267], SD: 357 mL). CI, confidence intervals; SD, standard deviations.

**FIG. 7.**
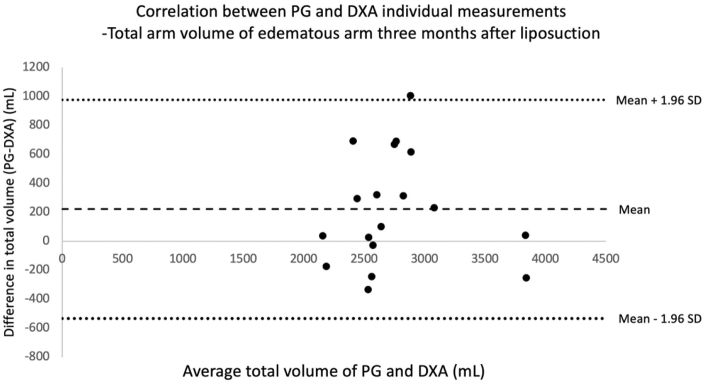
Bland–Altman correlation table showing correlation between total arm volumes in the lymphedematous arm 3 months after liposuction, measured with DXA and PG. Each point in the diagram represents one patient and two volume measurements, one with DXA and the other with PG. On the x-axis is the mean volume of the two measurements, and the y-axis represents the difference between the two measurements. The area between the two *dotted lines* represents the mean difference within 1.96 SD above and below the mean difference (*dashed line*). All measurements are in milliliters (mL). (mean = 222 mL [95% CI: 31–413], SD: 385 mL).

**FIG. 8.**
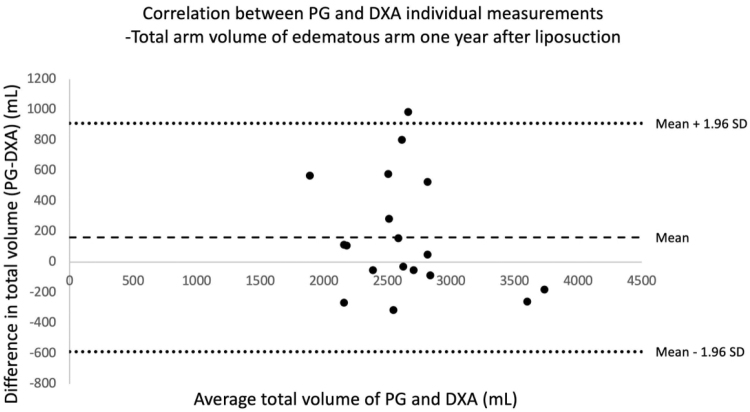
Bland–Altman correlation table showing correlation between total arm volumes in the lymphedematous arm 1 year after liposuction, measured with DXA and PG. Each point in the diagram represents one patient and two volume measurements, one with DXA and the other with PG. On the x-axis is the mean volume of the two measurements, and the y-axis represents the difference between the two measurements. The area between the two *dotted lines* represents the mean difference within 1.96 SD above and below the mean difference (*dashed line*). All measurements are in milliliters (mL). (mean = 162 mL [95% CI: *−*28 to 352], SD: 382 mL).

The correlation between volumes obtained from PG and DXA measurements is illustrated in Bland–Altman plots (Figs. 6–8). The mean (±standard deviations [SD]) preoperative difference in volume of the lymphedematous arm obtained with PG compared with DXA in 18 patients was 89 ± 357 mL (range: −503 to 829) (95% CI: −89 to 267), demonstrating no statistically significant difference. At most, the difference in these measurements was an overestimation in one patient of 829 mL using PG compared with DXA (4805 and 3976 mL, respectively). The expected upper 97.5 percentile (mean +1.96 SD) is 789 mL, meaning that 95% of all measured values will differ by up to 789 mL between the two methods.

At 3 months after liposuction, the mean (±SD) difference between PG and DXA was 222 ± 385 mL (range: −335 to 1005) (95% CI: 31–413), with an expected upper 97.5 percentile of 977 mL. Thus, the analysis indicates a statistically significant discrepancy between PG and DXA. The maximum difference between PG and DXA was 1005 mL in one patient (3385 and 2380 mL, respectively).

After 1 year, there was a mean(±SD) difference of 162 ± 382 mL (range: −316 to 984) (95% CI: −28 to 352) between measurements, with a maximal volume difference of 984 mL in one patient (3155 and 2171 mL, respectively) and an expected upper 97.5 percentile of 911 mL (Fig. 8). Thus, at this point, the difference between the methods is not statistically significant.

## Discussion

The postoperative course of tissue composition in lymphedematous arms was analyzed in 18 women with BCRL after liposuction in combination with CCT. In a previous study, we showed that the amount of fat, muscle, and bone mineral increased in lymphedematous arms.^9^ The reason for the increase in muscle and bone is hypothetically due to the increased load of the arm, which stimulates muscle and bone growth in a way similar to physical exercise. After liposuction, the amount of fat in the lymphedematous arm decreased during follow-up. At 1 year, a complete reduction was seen in excess volume measured with PG, with a slight overcorrection. These results were anticipated due to the overall satisfactory results of this treatment.

Although there was no change in BMI during follow-up, it might be interesting to include total body weight in a longer follow-up, to evaluate whether liposuction accentuates fat depositions in another part of the body, as seen in patients undergoing abdominal lipectomy due to obesity.^51^ In agreement with our previous findings, muscle volumes continued to decrease as the weight of the arm decreased.^6^ The volume of bone minerals showed a modest but statistically significant reduction after 1 year, but not after 3 months. We believe this may be due to a slower remodeling of bone in response to changes in load, the relatively small changes observed in bone volume, or the small sample size in this study.

There is a tendency for PG to measure somewhat higher volumes than DXA, which is illustrated in Bland–Altman plots. However, the difference in the measurements is only statistically significant in the follow-up at 3 months. The Bland–Altman plots comparing PG and DXA volumes of the nonlymphedematous arms are not included in this script, since the within-subject differences between the two measurements did not follow a normal distribution. A possible explanation for the different volumes obtained could involve a bias in the region of measurement, due to possible difficulties in targeting the exact same region in the different methods. The coefficients of variation within both measurements (2.5% for DXA^9^ and 0.61% for PG^43^) are relatively small, but cannot be discarded as having an influence on the discrepancy seen between PG and DXA measurements.

However, it should reasonably not influence the measurements in one direction giving the significant difference seen at 3 months. Converted to percentage of the total mean volume of both measurements, the average difference between PG and DXA preoperatively, 3 months after surgery, and 1 year after surgery was 2% (95% CI: −2 to 7), 8% (95% CI: 1–15), and 6% (95% CI: −1 to 13), respectively. The recommendation is, therefore, to take caution when using PG and DXA as interchangeable methods in terms of measuring total arm volumes in patients with lymphedema. They can be used separately as a means of following volumes in one patient. Although DXA exposes the patient to radiation,^52^ the radiation dose is very small, and we believe the extensive availability of DXA makes it a valuable method for evaluating tissue composition in lymphedematous arms.

Lean tissue includes all components in the arm except fat and bone. It includes muscle, fluid (lymph and blood), extracellular matrix, tendons, and skin, which are not separated in the analysis using DXA volumes. The lymph fluid is decreased to a minimum by CDT before surgery, resulting in non- (or minimal) pitting lymphedema, and blood volumes can be assumed to be approximately equal in both arms. When a tourniquet was used so that there was no tumescence fluid, analysis showed a median fluid volume of 0% (IQR: 0–8) in the aspirate. However, the volume of skin, tendons, and extracellular matrix might be increased in the lymphedematous arms and could possibly reduce postoperatively. Muscle volumes could, therefore, be lower than presented in this study.

There is a disagreement in the literature regarding the presence of muscle hypertrophy in limbs with lymphedema. DXA scans have previously been performed on 56 women with BCRL, showing a small but significant increase in lean volume in the affected arm compared with the nonaffected arm, but no significant difference in lean volume compared with the same arm (dominant or nondominant) in healthy controls.^53^ On average, the controls (*n* = 44) had 205 mL excess lean volume in the dominant side compared with the nondominant side. The 56 women with lymphedema had, on average, an excess volume of 274 mL in the lymphedematous arm. In this study, the participants had a median of 1425 mL excess volume. The more than five times larger excess volumes seen in lymphedematous arms in this study enable the possibility of muscle hypertrophy in the arm due to an increased load.

Furthermore, due to the large excess volume, the impact dominant and nondominant sides have on tissue composition will be reduced. Other studies evaluating arm lymphedemas with DXA have found a similar slight increase in lean mass in the affected arm compared with the nonaffected arm.^54–56^ The theory of increased muscle volume in lymphedematous limbs has also been investigated with MRI, which shows somewhat conflicting results. Either excess muscle volume has not been significantly found at all,^57,58^ only been found occasionally (5 out of 32 patients),^59^ or found in modest excess (median 959 mL muscle volume in the affected arm compared with 898 mL in the nonaffected arm in 5 patients).^38^

The volume of the subfascial compartment in 32 lymphedematous legs has also been investigated with CT, showing a significant increase (208 mL) in this compartment of the affected leg compared with the nonaffected leg.^60^ The authors highlight that further research has to determine which tissue in the subfascial compartment increases in leg lymphedemas. Heterogeneous patient selection with both early and late-stage lymphedemas as well as the magnitude of the lymphedema may affect the outcome in some studies. Two studies, using MRI to assess the same 13 patients with late-stage lymphedema (seven arms and six legs), showed an insignificant increase in water/muscle volume in the subfascial compartment of the affected limb, with a significant reduction after 3, 6, and 12 months.^6,39^ This, and the findings in this article, supports the theory that a decrease in load after liposuction reduces muscle hypertrophy.

Another source of potential errors in this study involves the densities used to convert the mass measured with DXA into volumes. There is consensus in the literature on the densities of muscle and adipose tissue, with minor variances in density depending on temperature and hydration level.^44–46^ In this article, the value corresponding to the density of fat at room temperature was chosen, given that the portions of aspirated fat and fluids are measured at room temperature 24 hours after surgery, when fat and fluid have separated in the canister.

The major discrepancy in the literature involves densities for bone mass, with values ranging from 1.7 to 3.2.^48,61–63^ The reason for this variation is probably the common use of DXA in measuring bone composition and diagnosing osteoporosis. In these cases, DXA is used to measure the mineral content in a designated area,^64^ and this is used to compare bone mineral composition between patients. The use of the term bone mineral density in bone densitometry is somewhat misleading. The expression refers to the quantity of bone mineral in a defined area (cm^2^) of imaging, rather than the actual density of bone mineral. This differs from the measurements in this study, where total BMC is used, representing the total amount of bone mineral mass in grams within the scanned region.

Therefore, the density for hydroxyapatite^65^ is used in this study to calculate bone volumes, and is expected to have some margin of error since this is the main but not the only element in bone mineral. However, bone mineral volume in the arms only constitutes a small proportion of the total volumes, and small differences in bone density will not be crucial to the study outcomes.

## Conclusions

The previously reported preoperative excess fat volume of 73% in these 18 patients declined to a complete reduction at 3 months, and these results were maintained after 1 year. The DXA analysis also showed a decrease in the previously reported preoperative excess muscle volume of 54% in the lymphedematous arm. Although a significant reduction in bone volumes could be seen after 1 year, this finding should be interpreted with caution, due to its exploratory nature and the relatively low significance level. The differences between DXA and PG in terms of volume measurements are of a level that has important clinical implications. These methods should, therefore, not be used interchangeably to measure total volumes, but can be used separately.

Further studies could investigate the different components of lean tissue mass, focusing on changes in muscle volume in lymphedemas before and after liposuction, and with imaging modalities that enable muscle tissue to be separated from other lean tissue. In conclusion, liposuction is an effective method for removing excess adipose tissue in chronic nonpitting BCRL, as results improve continuously during the first 12 months after treatment.
